# Clinical effects and treatment outcomes of long-term compulsory in-patient treatment of treatment-resistant patients with severe mental illness and substance-use disorder

**DOI:** 10.1186/s12888-019-2254-9

**Published:** 2019-09-03

**Authors:** G. D. van Kranenburg, R. H. S. van den Brink, W. G. Mulder, W. J. Diekman, G. H. M. Pijnenborg, C. L. Mulder

**Affiliations:** 1 Drenthe Mental Health Organisation, P.O. Box: 30007, 9400 RA Assen, Sustainable Residence, Beilen, The Netherlands; 2 University of Groningen, Department of Psychiatry, University Medical Center Groningen, Rob Giel Research Center, Groningen, the Netherlands; 3 Bouman GGZ, Rotterdam, the Netherlands; 4 Addiction Service North Netherlands, Groningen, the Netherlands; 5 Drenthe Mental Healthcare Organisation, Department of Psychotic Disorders, Assen, Department of Clinical, Psychology and Experimental Psychopathology, Faculty of Behavioural and Social Science, University of Groningen, Groningen, the Netherlands; 6 Epidemiological and Social Psychiatric Research Institute, Department of Psychiatry, Erasmus MC, Rotterdam, the Netherlands

**Keywords:** Severely mentally ill (SMI), Dual diagnosis, Compulsory treatment, Treatment resistant, Routine outcome assessment

## Abstract

**Background:**

The treatment of homeless dual-diagnosis patients (i.e., those with severe mental illness and substance-use disorder) is difficult and often fails. For patients in the Netherlands who had not responded to earlier voluntary and compulsory treatment, a new treatment facility – Sustainable Residence (SuRe) – was developed to offer long-term compulsory in-patient treatment.

**Aim of the study:**

To study patterns of changes in clinical and functional outcomes during treatment at SuRe and how these relate to eventual treatment outcome.

**Methods:**

On the basis of the intensity of care needed after four years, three groups of patients were distinguished (total *n* = 165): those discharged to a less restrictive and less supportive setting (*n* = 70, 42%), those still hospitalized at SuRe at the end of the four-year study period (*n* = 69, 42%) and those referred to a more appropriate setting (*n* = 26, 16%). Random coefficient analysis was used to examine differences between groups regarding changes in clinical and functional outcomes during treatment. During treatment, outcomes were monitored using Routine Outcome Assessment.

**Results:**

All three groups made small but significant improvements on global psychosocial functioning, distress and therapeutic alliance (effect sizes (ES) 0.11 to 0.16 per year). Patients who were discharged to a less restrictive setting showed small to moderate improvement in risk to self and others, psychiatric symptoms, and skills for daily living (ES 0.19–0.33 per year and 0.42–0.73 for their mean 2.2-year treatment period). Patients remaining at SuRe showed a small increase in risk to self (ES 0.20 per year; 0.80 for their treatment period of four years or more). Oppositional behaviour was consistently greater in referred patients than in the other groups (ES 0.74–0.75).

**Conclusion:**

Long-term compulsory treatment appeared to have helped improve clinical and functional outcomes in a substantial proportion (42%) of previously severely dysfunctional, treatment-resistant dual-diagnosis patients, who could then be discharged to a less restrictive and less supportive environment. However, risk-to-self increased in a similar proportion. A smaller number of patients (16%) showed marked oppositional behaviour and needed a higher level of care and protection in another facility.

## Background

Treatment resistance and disengagement from mental-health services are major obstacles in the treatment of difficult-to-engage patients with a dual diagnosis (i.e., those with Severe Mental Illness (SMI) and substance-use disorder). About 50% of these patients do not respond well to integrated outpatient services [1], partly because they lack stable, safe and supportive living arrangements. Many of them are homeless or live in neighbourhoods that are affected by drug abuse. Similarly, as outpatient services for difficult-to-engage SMI patients vary widely between the large European cities, and as the overall quality of these services is poor [2], many patients live on the streets, where they cause nuisance and lack mental health care. As a consequence of untreated psychotic symptoms and addiction problems, they are at risk of victimization, somatic illnesses and imprisonment for violent behaviour [2].

There is evidence that long-term residential dual-diagnosis programmes can be effective for dual-diagnosis patients who have not responded to outpatient treatment [3]. However, if these programmes are voluntary, their attrition rate can be as high as 75% [1]. Long-term compulsory treatment may be an alternative for patients who need mental healthcare and are a risk to themselves or others, but continuously drop out of voluntary programmes.

### Sustainable residence (SuRe): long-term compulsory treatment

Sustainable Residence (SuRe) was developed in the Netherlands as a purpose-built long-term compulsory treatment facility. It is intended for homeless dual-diagnosis patients who are seen as treatment-resistant by the existing services, as they have a long history of treatment efforts (including multiple compulsory admissions) which did not lead to lasting improvements. [4]. Due to their worrisome physical and psychological state, the patients posed a lasting risk to themselves and/or others, and usually caused serious public nuisance.

Establishing a new structure to provide long-term involuntary care has been controversial from the start of SuRe. Such provision is at odds with reforms in mental health care and the United Nations Convention of the Rights of Persons with Disabilities, which aim to reduce coercion and to maximize patient autonomy, among other things. A strong need was felt, however, to avert the grave risk of self-destruction these patients run, and to improve their quality of life. A treatment option such as SuRe was therefore deemed necessary, as a last resort: an ‘ultimum remedium’.

Located in the northern Netherlands, this new and unique treatment facility involves the collaboration of a mental health service and an addiction service. It opened in 2007. Patients are referred on the basis of involuntary admission by the municipal health services of three major Dutch cities. They are admitted for as long as necessary on the basis of a court order. To obtain such order, an independent psychiatrist makes an assessment which is requested by the treatment provider. This assessment is sent to the judge, who decides on an extension of the order every six or twelve months.

Patients in the Netherlands can be admitted involuntarily only if they have been diagnosed with a mental disorder that makes them an actual or potential danger to themselves and/or others. Their involuntary admission is permitted only if they are unwilling to be admitted voluntarily and if there is no other recourse. This legal context applies to all mental health facilities in the Netherlands. However, when SuRe was established, the context of patients admitted to it was discussed with the District Attorney, particularly with regard to their history of involuntary admissions to psychiatric hospitals, their failure to improve during treatment, and their history of neglect and trauma. It was agreed that account of this context would be taken by judges issuing new court orders.

Although treatment at SuRe focuses on improving patients’ functioning and quality of life, the extent to which functional recovery was possible was unclear when SuRe opened.

### Aim of the study

To investigate the relationship between clinical changes during treatment and eventual treatment outcomes of long-term involuntary in-patient treatment of previously treatment-resistant dual-diagnosis patients.

## Methods

### Design and setting

This observational study included all patients hospitalized at SuRe between August 2009 and January 2015. Patients’ clinical and functional outcomes were monitored during their treatment at SuRe, over a maximum period of four years after their admittance. We excluded patients who were still in treatment at SuRe in January 2015 but had not yet been in treatment for four years.

There are four criteria for admission to SuRe: (1) dual diagnosis (SMI and substance-use disorder); (2) a history of homelessness; (3) failure of earlier treatment to achieve lasting improvement, despite the use of appropriate means, including multiple compulsory admissions; and (4) the imposition of a civil-law court order for involuntary admission on the grounds of the risk of lasting danger to self or others.

The main criterion for discharge from SuRe is a reduction in the risk to oneself and/or others.

Over a four-year period we used Routine Outcome Assessment (ROA) to monitor developments in patients’ clinical and functional outcomes during their hospitalization at SuRe. ROA was part of standard care; it consisted of periodic ratings of patient functioning by the patient’s case manager or by the treatment provider who had principal responsibility for the patient’s treatment.

The study protocol was reviewed and approved by the Dutch Medical Ethical Committee for the Mental Health Services and judged to be in accordance with the Dutch Medical Research Involving Human Subjects Act (Metc no. NL30019.097.09).

### Study groups

On the basis of treatment facility at the end of the four-year study period, we distinguished three study groups of patients post hoc.

The first group, the Discharged group, consisted of patients for whom treatment at SuRe had been successful. These patients had been discharged to a less restrictive and less supportive setting, as exemplified by the legal basis for admittance to the facility and its policy with respect to leave, autonomy and the support provided to patients. The second group, the Continued Care group, consisted of patients who were still hospitalized at SuRe after a minimum of four years because their treatment was not yet complete. The third group, the Referred group, consisted of patients for whom treatment at SuRe had been unsuccessful. They had to be referred to a more restrictive setting (usually a closed forensic psychiatric treatment facility) or a more appropriate one (usually a facility for the mentally disabled).

### Treatment

Treatment at SuRe is comprehensive and highly supportive. It is delivered by multidisciplinary teams consisting of a psychiatrist, psychologist, case managers, residential supervisors and domestic workers. The case managers are the principal treatment providers. They are nurses or social workers with a Bachelor’s degree who coordinate the patients’ treatment and have an overview of their condition and treatment, and also of the patients’ social networks.

Other disciplines are also available: a somatic doctor, nurses, social workers, creative therapists, psychomotor therapists, social juridical workers, activity supervisors and a cultural anthropologist. All patients in SuRe’s admission ward have a room of their own. The same complex also has a crisis unit and a small unit for long-term intensive care. When they have stabilized, patients move to their own house in a closed area that was designed according to the principles of a ‘healing environment’. If their condition worsens, they can be referred to the crisis unit for a short period or to the unit for intensive care for a longer period. SuRe has a maximum capacity of 133 patients.

### Assessment of patients’ clinical and functional outcomes

The patients had been admitted to SuRe on the basis of a civil-law court order stating that a psychiatric disorder had caused them to pose a risk to themselves or others.

Risk to oneself or others was assessed with the Risk Assessment Checklist (RAC) [5], a Dutch rating scale developed for psychiatrists to rate patients who have been committed by court order to compulsory treatment order under Dutch civil law. The RAC rates seven types of risk on a five-point scale (from 0 ‘no risk’ to 4 ‘high risk’). Four types of risk are classified as a ‘risk to oneself’: (1) the risk of committing suicide or otherwise harming oneself, (2) the risk of social deterioration, (3) the risk of serious self-neglect and (4) the risk of invoking aggression due to disruptive behaviour. Similarly, three types of risk are classified as ‘risks to others’: (1) the risk of killing or seriously harming someone, (2) the risk of damaging the mental health of others, and (3) the risk to the general safety of persons or goods. Separate sum scores are calculated for the risk-to-self items and the risk-to-others items, with higher scores indicating greater risk. The RAC is rated by the provider who has principal responsibility for the patient’s treatment.

In addition to the risk to oneself or others, the following aspects of patient functioning were assessed: psychiatric and addiction symptoms; somatic, cognitive and psychosocial functioning; skills for activities of daily living; and the patient’s working relationship with treatment providers. These aspects of patient functioning were assessed using the following five instruments:

(1) The split version of the Global Assessment of Functioning (GAF) [6] was used as an overall measure of patient functioning. This instrument has separate scales for Symptoms and Disability. Ratings are made on scales ranging from 0 to 100, with a higher score indicating better patient functioning. The GAF was filled out by the treatment provider who had principal responsibility for the treatment.

(2) Symptoms and psychosocial functioning were assessed using the Health of the Nation Outcomes Scale (HoNOS) [7]. The HoNOS is used to assess adult mental-health patients’ behavioural problems (including addiction), disabilities, psychiatric symptoms and social behaviour over the last two weeks. It consists of twelve observer-rated items, each using five points from 0 (no problems) to 4 (severe/very severe problems). Four subscale scores are calculated by summing item scores: Behaviour (3 items, e.g., overactive or agitated behaviour, problem drinking or drug-taking); Impairments (2 items, e.g., cognitive problems); Symptoms (3 items, e.g., depressed mood and problems associated with hallucinations and delusions); and Social (4 items, e.g., problems with relationships, and problems with activities of daily living). In addition, a total score is calculated by summing all items. Higher scores on all scales indicate worse patient functioning. The HoNOS was rated by the patient’s case manager on the basis of inputs from the team that provided the patient’s daily care and residential supervision.

(3) Activities of daily living and disruptive behaviour were assessed using the Forensic Inpatient Observation Scale (FIOS) [8], which was developed to assess the functioning of forensic patients. It is divided into six subscales, one of which was omitted (i.e., Insight) because the items refer to offences committed by forensic patients. The remaining five scales are (1) Self-care (7 items, e.g., brushing teeth, and cleaning their room/house); (2) Social behaviour (6 items, e.g., joining the group for activities, and starting a conversation); (3) Oppositional behaviour (10 items, e.g., verbal aggression, and lying); (4) Verbal skills (3 items, e.g., understanding others, and talking intelligibly); and (5) Distress (5 items, e.g., feelings of hopelessness, and wishing to be dead). Quantitative ratings were made through 5-point Likert scales (1 = never to 5 = always) on the basis of patient behaviour over a three-week period. Higher rates indicate worse patient functioning. The FIOS was rated in combination with the HoNOS by the case manager and the team of daily care providers.

(4) Cognitive aspects of patient functioning were assessed using the Executive Observation Scale (EOS), a translation of the observation list developed by Pollens [9]. It consists of eight items covering cognitive and behavioural aspects of executive functioning in everyday tasks: i.e., awareness, planning, goal setting, self-initiation, self-inhibition, self-monitoring, and ability to change set and strategic behaviour. Each item is rated on a Likert scale from 1 (= complete inability) to 4 (= complete independence and ability). A single sum score is calculated, with higher scores indicating better cognitive functioning. The EOS was rated by the case manager and the team of daily care providers.

(5) Because patients at SuRe have a history of disengagement from care, we added the Helping Alliance Scale [10] in order to assess the quality of the therapeutic alliance between the patient and treatment providers. The scale has two versions: a client version and a therapist version. For this study we used the therapist version (HAS-T), which captures the therapist’s view of his or her therapeutic alliance with the patient. It is a short 5-item scale covering basic elements of a therapeutic relationship, e.g., understanding the patient, and feeling actively involved and able to treat him/her. These items are rated on 10-point scales. A sum score of the five items is calculated, with a higher score indicating the therapist’s opinion that the therapeutic relationship is better. The HAS-T was rated by the case manager.

Once a year all the scales per patient were administered together. The HoNOS and FIOS were rated twice more per year.

### Analysis

Our analyses focused on the differences between the three groups with regard to changes in clinical and functional outcomes over four years. First, to test group differences regarding baseline characteristics (i.e., upon admission to SuRe), we used Chi-square tests for categorical characteristics and analysis of variance for continuous characteristics.

Then, to examine differences between the study groups with regard to patients’ clinical and functional outcomes over the four-year period, we used random coefficient analyses – a specific type of linear mixed models that takes account of the dependency of repeated observations obtained from the same individual over time and is therefore suitable for performing regression analyses with repeated-measures data. This method also includes subjects regardless of the number and timing of assessments over the study period. For each clinical or functional outcome separately, we tested whether there is (1) a main effect of time (i.e., a linear increase or decrease in patient outcome for all study groups over the four-year study period); (2) a time-independent main effect of group (i.e., a stable difference in patient outcome between the study groups); or (3) an interaction between time and group (i.e., differences between study groups in the linear development of patient outcome over time). To determine the best-fitting random coefficient model, the likelihood ratio test was used to compare models with random coefficients for intercept and/or slope per subject. All tests used a significance level of α = .05. Effect sizes were standardized by dividing the effect on a patient outcome measure (either the difference between group means or the change in outcome per year) by the overall standard deviation for that specific outcome on the first assessments per person.

## Results

Two hundred and twenty-nine patients were hospitalized at SuRe between August 2009 and January 2015. Sixty-four of them (27.9%) were excluded from the present study: fifty because they had not yet received four years of treatment, seven because they died at SuRe before the first four years had elapsed, five because they absconded from SuRe within their first four years, and two because they were imprisoned during their first four years at SuRe for crimes they had committed before their admission. With regard to the baseline characteristics listed in Table 1, those excluded did not differ significantly from those included.

**Table 1:** Baseline characteristics of study groups of dual-diagnosis patients admitted to Sustainable Residence

Characteristic	Total (*N* = 165)	Group A^a^ (*N* = 70)	Group B^a^ (*N* = 69)	Group C^a^ (*N* = 26)	Χ^2^/F	p	Group Differences^b^
Gender (% male)	84,2	78.6	87.0	92.3	3.04	.23	
Age (mean in years, sd)	39.2 (8.2)	40.4 (8.6)	40.0 (7.8)	33.8 (5.7)	7.29	<.01	C < A; C < B
Education^d^ (%)							
low	50.9	54.3	50.7	42.3	1.69	0.82	
intermediate	23,0	21.4	27.5	15.4			
high	7.9	5.7	10.1	7.7			
missing	18.2	18.6	11.6	34.6			
Country of birth (%)							
Netherlands	44.2	42.9	44.9	46.2	3.03	0.22	
other^**c**^	49.1	52.9	55.1	23.1			
missing	6.7	4.3	0.0	30.8			
Lived with a partner (%)							
yes	30.9	40.0	26.1	19.2	7,77	0.02	A > B
missing	31.5	34.3	24.6	42.3			
Diagnosis on Axis I (%)							
psychotic disorder	90.9	87.1	95.7	88.0	3,57	0.20	
substance abuse	93.9	92.9	95.7	92.0	3.35	0.19	
Diagnosis on Axis II (%)							
personality disorder	36.4	31.4	39.1	42.3	1.93	0.38	
borderline intellectual functioning	15.8	15.7	14.5	19.2	1.13	0.55	
missing	12.7	15.7	5.8	23.1			

^**a**^ Group A: Discharged group; Group B: Continued Care group; Group C: Referred group

^**b**^ Differences between groups significant at .05 level

^**c**^ Countries on the following continents: South America (22.4%), Africa (12.1%), North America (8.5%), Asia (3.6%),

Europe (1.8%), Oceania (0.6%)

^d^ Low: elementary school or less; intermediate: low-level/intermediate level secondary school; high: high level secondary

school, intermediate vocational or higher education

The final study sample consisted of a hundred and sixty-five patients, seventy of whom (42.4%) had been discharged to a less restrictive and supportive mental health facility within four years of admission to SuRe (the Discharged group); sixty-nine of whom (41.8%) were still in treatment at SuRe at the end of the four-year study period (the Continued Care group); and twenty-six of whom (15.8%) had been referred to a more appropriate or more restrictive facility during the four-year period (the Referred group).

With regard to the types of care, Discharged patients moved to one of the following: an open psychiatric ward, supported housing, supported independent living, or independent living with treatment by Assertive Community Treatment teams. Patients in the Referred group were referred either to a more restrictive setting (i.e., a closed psychiatric treatment facility or a forensic psychiatric treatment facility) or to a more appropriate setting (i.e., a treatment facility for the mentally disabled).

Except for patients in the Continued Care group, who by definition had stayed for four years or more, the mean hospital stay at SuRe was 2.2 years (SD = 1.1) in the Discharged group, and 0.9 (0.7) years in the Referred group (T = 6.46, *p* < .01).

Table 1 shows the baseline characteristics of the three study groups, which differed significantly at baseline with regard to the patients’ ages and histories of living with a partner. Those in the Referred group had a significantly lower mean age than those in the Discharged and Continued Care groups. And whereas more Discharged patients than Continued Care patients had ever lived with a partner, the difference with Referred patients did not reach significance, due probably to the small number of patients in the Referred group.

Table 2 shows the differences between study groups with regard to developments in patient functioning in the period studied. Main effects for group and time were tested, as were the interaction between group and time (as indicated in the top row of the table).

**Table 2:** Effects of group, time, and their interaction on clinical and functional outcomes of dual-diagnosis patients admitted to Sustainable Residence

Outcome	Number of assessments	Scale	Interaction Group X Time		Group effect			Time effect					
			F	p	F	p	Group differences^**a,b**^	In group^**a**^	Change/yr (se)	95% CI	F/t	p	Group differences^**a,b**^
HoNOS^c^	540	Behaviour	0.61	.55	9.28	<.01	A < B < C	A-C	−0.11 (0.11)	−0.33; 0.10	1.08	.30	
		Impairment	0.35	.71	0.36	.70		A-C	−0.07 (0.08)	− 0.24; 0.09	0.82	.37	
		Symptoms	5.84	<.01	3.19	.04	B < C	A B C	−0.66 (0.21) 0.18 (0.13) −1.18 (1.67)	−1.07; − 0.24 − 0.08; 0.45 −4.45; 3.27	3.11 1.36 0.71	<.01 .18 .48	A < B
		Social	1.47	.23	0.03	.97		A-C	−0.42 (0.16)	−0.72; − 0.11	7.21	<.01	
		Total	1.59	.21	2.60	.08		A-C	−0.68 (0.34)	−1.34; − 0.01	3.95	.05	
FIOS	532	Self-care	6.63	<.01	2.35	.10		A B C	1.14 (0.31) − 0.13 (0.20) −2.02 (1.82)	0.53; 1.75 − 0.51; 0.26 −5.59; 1.55	3.64 0.65 1.11	<.01 .52 .27	A > B
		Social behaviour	1.75	.18	0.21	.82		A-C	0.28 (0.23)	− 0.18; 0.73	1.40	.24	
		Oppositional behaviour	1.96	.15	3.25	.04	A > C; B > C	A-C	0.29 (0.30)	−0.29; 0.87	0.94	.34	
		Verbal skills	4.47	.01	1.89	.16		A B C	0.68 (0.17) 0.05 (0.12) 0.61 (0.77)	0.35; 1.01 −0.19; 0.30 − 0.90; 2.11	4.00 0.44 0.79	<.01 .66 .43	A > B
		Distress	2.02	.13	1.56	.21		A-C	0.37 (0.12)	0.14; 0.61	9.67	<.01	
GAF	288	Symptoms	7.43	<.01	3.93	.02	B > A	A B C	3.15 (0.91) −0.96 (0.64) −2.51 (2.75)	1.38; 4.93 −2.21; 0.29 −7.91; 2.89	3.48 1.50 0.91	<.01 .14 .36	A > B
		Disability	3.99	.02	0.90	.41		A B C	2.17 (0.61) 0.20 (0.36) 0.14 (1.96)	0.99; 3.35 −0.50; 0.91 −3.71; 3.99	3.61 0.56 0.07	<.01 .58 .94	A > B
RAC^c^	286	Risk to self	7.39	<.01	5.64	<.01	B < A; B < C	A B C	−0.15 (0.06) 0.11 (0.04) − 0.05 (0.19)	−0.26; − 0.03 0.04; 0.18 − 0.42; 0.32	2.56 3.11 0.27	.01 <.01 .79	A < B
		Risk to others	3.82	.02	2.75	.07		A B C	−0.15 (0.06) 0.00 (0.04) 0.29 (0.19)	−0.26; − 0.03 − 0.07; 0.07 − 0.09; 0.66	2.55 0.02 1.50	.01 .98 .13	A < B; A < C
HAS-T	207	Total	0.78	.46	0.93	.40		A-C	0.17 (0.07)	0.04; 0.30	6.27	.01	
Executive functioning	248	Total	2.71	.07	0.76	.47		A-C	0.01 (0.02)	−0.03; 0.05	0.66	.51	

^a^ Group A: Discharged group; Group B: Continued Care group; Group C: Referred group

^b^ Differences between groups significant at .05 level

^c^ Contrary to the other outcomes, higher scores on these outcomes indicate worse patient functioning

Differences between the study groups with regard to developments in patients’ functioning (i.e., an interaction between group and time, as tested in columns 4 and 5 of the table) were found on the Symptoms subscale of the HoNOS, on the Self-care and Verbal Skills subscales of the FIOS, on the Symptoms and Disability scales of the GAF, and on the Risk to Self and Risk to Others subscales of the RAC.

On all these scales, the Discharged group (i.e., the patients discharged to less intensive care (group A in Table 2)) showed improvements in functioning between yearly assessments. In columns 10 to 13 of the table, these developments over time are shown and tested for the study group (or combination of groups) indicated in column 9; in the last column of the table they are compared between groups. Based on the standard deviation (shown below in brackets), the effect sizes of these improvements per year were as follows: 0.24 per year for HoNOS-Symptoms (SD = 2.73); 0.19 for FIOS-Self-care (5.87); 0.31 for FIOS-Verbal skills (2.17); 0.33 for GAF-Symptoms (9.52); 0.33 for GAF-Disability (6.51); 0.27 for Risk to Self (0.56); and 0.24 for Risk to Others (0.62). In contrast, patients in the Continued Care group (i.e., patients who remained at SuRe (group B in Table 2)), showed a decline in patient functioning regarding Risk to Self, with an effect size of 0.20 per year. In combination with the above interaction effects, differences between groups at admission to SuRe (shown and tested in columns 6 and 7 of Table 2) were found on HoNOS-Symptoms, GAF-Symptoms, and Risk to Self. As indicated in column 8 of Table 2, the Continued Care group showed better patient functioning at admission than the Referred group (i.e., patients referred to a more appropriate or more restrictive setting (group C in Table 2)) on the HoNOS-Symptoms scale (effects size 1.00), and better functioning than the Discharged group on the GAF-Symptoms scale (0.86), and better than both the Discharged and Referred groups on Risk to Self (0.84 resp. 1.18).

Patient functioning improved equally in all study groups (i.e., a main effect of time was found) on the HoNOS-Social subscale (effect size 0.14 per year; SD = 3.00); on the HoNOS-Total scale (0.11; 6.39); the FIOS - Distress subscale (0.12; 2.97); and the HAS-T scale (0.16; 1.05).

Finally, differences in functioning between the groups were stable over the entire study period (i.e., main effect of group) on the HoNOS-Behaviour subscale, with the Discharged group showing significantly better functioning than the Continued Care group (effect size 0.30; 2.10); and with both the Discharged and Continued Care groups showing better functioning than the Referred group (effect sizes 1.26 and 0.97, respectively). Similarly, on the FIOS-Oppositional Behaviour subscale, the Discharged group (0.74) and Continued Care group (0.75) showed better functioning than the Referred group (SD = 6.32).

There was no effect of group, time or group-time interaction on the HoNOS-Impairment subscale, the FIOS-Social behaviour subscale, or the Executive Observation Scale.

## Discussion

In this study we evaluated long-term compulsory in-patient treatment for difficult-to-engage dual-diagnosis patients. When they were referred to SuRe, all patients were considered to be treatment resistant. Until this study, the effects of long-term compulsory treatment were unknown.

Our first important finding is that marked improvements in functioning were possible in this group. Due to improvements in functioning, a substantial number of patients (42%) could be discharged within four years and referred to less restrictive and less supportive facilities.

Our results also revealed meaningful differences in patient functioning and in improvements in functioning between patients who were discharged, those in continued care, and those who were referred to another treatment setting during treatment at SuRe.

Patients who were discharged to a less restrictive and supportive setting (such as supported housing) showed not only a decrease in risk to themselves and others, but also improvements in psychiatric symptoms, self-care behaviour, verbal skills and disability, with effect-sizes ranging from 0.19–0.33 per year. Although these effects may seem small, it should be remembered that the mean duration of treatment in this patient group was 2.2 years. Over the entire treatment period, the size of these effects was medium large (0.42–0.73). These improvements in functioning may have been instrumental to these patients’ referral to regular mental healthcare facilities. The Discharged group also showed better functioning on the HoNOS-Behaviour subscale than the other groups, and scored better on the FIOS-Oppositional Behaviour subscale than the Referred group. These differences were already present at admittance to SuRe, and remained during treatment. These findings may be useful to clinicians not only in shaping their expectations of treatment and attuning their treatment approach, but also in selecting patients for SuRe.

Although patients who remained at SuRe had started out with lower scores on psychiatric symptoms and risk to themselves than patients in the two other groups, their risk to themselves increased during treatment. The size of this effect was 0.20 per year. While this may raise questions about the effectiveness of the treatment for these patients, suggesting that the treatment had an iatrogenic effect, functional deterioration is often integral to the natural course of chronic psychosis – a deterioration that may have been exacerbated by the combination with long-term substance abuse. Patients’ awareness of these effects in clinical surroundings might also reduce their motivation for treatment, and increase demoralization and risk to self. At the very least, these findings signal that this essential aspect of patient functioning should be carefully monitored. The reason for this increase should also be investigated, as it may be crucial to decisions on extending or not extending treatment at SuRe.

From admission on, patients who were later referred to a more restrictive or more appropriate facility – in most cases a forensic one – had shown substantially more behavioural problems and oppositional behaviour (effect size 0.74 to 0.75) than other patients during their treatment at SuRe. This may be an important issue to consider when selecting patients for Sure. In all cases, seriously dangerous behaviour (such as gravely harming a supervisor) was the reason for their referral to another setting. As the principal treatment approach at SuRe is highly supportive, the question is whether these patients actually need a more structured treatment approach and a more restrictive setting, inside SuRe or elsewhere.

Although no improvements on the related measures of impairment and social behaviour could be established, we did find improvements during treatment for all patients regarding therapeutic alliance as perceived by the treatment provider, distress and psychosocial functioning. The fact that we found no improvements on executive functioning may indicate that cognitive problems are lasting results not only of a psychotic disorder, but also of a way of life characterized by self-neglect and long-term substance abuse.

Unlike with long-term voluntary treatment, there was hardly any dropout from this patient group, even though patients were permitted to go on leave. Only five patients failed to return after leaving SuRe.

### Limitations

Even though the development of patient functioning was modelled in a linear fashion, progress during the treatment period may not be a linear process. But as the number of assessments gathered by ROA was limited for some outcomes, we preferred – for reasons of parsimoniousness – the simplification of treatment progress that linear modelling provided. Neither did we assess outcomes at fixed intervals or equally frequently for each patient. This was a consequence of the way routine outcome assessment takes place in everyday practice. However, the method of analysis we chose can accommodate these peculiarities of the data we gathered.

A second limitation is the absence of a control group, which lay in the impossibility of finding a suitable patient group or location. From both scientific and practical points of view, the most informative option seemed to be to compare subgroups that clearly differed in terms of the success of their treatment or of their ‘suitability for treatment at SuRe’. Inevitably, however, a risk of circular reasoning was introduced by comparing developments in patients’ functioning between groups that were selected on the basis of their treatment outcome, or, more precisely, of their referral to other services. The advantage of our approach however, is that it identified the developments in specific aspects of patient functioning that are related to – and may also be instrumental to – ultimate treatment and referral outcomes.

## Conclusions

The treatment at SuRe has been controversial from the start: patients are admitted involuntarily to a treatment facility for a length of time that is at odds with the reforms in mental healthcare. The coercive nature of the treatment has received particular criticism. Nonetheless, a substantial proportion of severely dysfunctional dual-diagnosis patients (42%), all of whom had been considered to be treatment resistant, improved during long-term compulsory treatment, and could be discharged to less restrictive and supportive facilities such as supported housing. Given these results, treatment at SuRe is an alternative that enables patients who drop out of voluntary programmes to avoid ultimate physical and social deterioration.

It would be of great clinical value if it were possible to predict with some certainty whether or not a patient belongs to this group.

## Data Availability

Data are available on request.

## Abbreviations

EOS: Executive Observation Scale
FIOS: Forensic Inpatient Observation Scale
GAF: Global Assessment of Functioning
HAS: Helping Alliance Scale
HoNOS: Health of Nation Outcome Scale
RAC: Risk Assessment Checklist
ROA: Routine Outcome Assessment
SMI: Severe mental Illness
SuRe: Sustainable Residence
